# Development and Validation of the Prognostic Index Based on Inflammation-Related Gene Analysis in Idiopathic Pulmonary Fibrosis

**DOI:** 10.3389/fmolb.2021.667459

**Published:** 2021-07-22

**Authors:** Yanjiao Lu, Jinkun Chen, Kun Tang, Shanshan Wang, Zhen Tian, Meijia Wang, Jianping Zhao, Jungang Xie

**Affiliations:** ^1^ Department of Respiratory and Critical Care Medicine, National Clinical Research Center of Respiratory Disease, Tongji Hospital, Tongji Medical College, Huazhong University of Science and Technology, Wuhan, China; ^2^ Department of science, Western University, London, ON, Canada

**Keywords:** idiopathic pulmonary fibrosis, inflammation, prognostic, interstitial lung disease, gene

## Abstract

**Background:** Historically, idiopathic pulmonary fibrosis (IPF) was considered a chronic inflammation disorder, but this conception was reassessed in the past decades. Our understanding of the role of inflammation in IPF and its association with clinical significance remained incomplete.

**Methods:** We downloaded mRNA expression data of peripheral blood mononuclear cells (PBMCs) from the Gene Expression Omnibus (GEO) repository. Inflammation-related genes (IRGs) expressed differently between IPF and control (CTRL) were determined. In this study, we systemically analyzed the expression of differently expressed IRGs by comprehensive bioinformatic analysis, and then investigated their potential prognostic values. The related prognostic gene expressions were verified in our cohort.

**Results:** 110 differently expressed IRGs were identified in this study, including 64 upregulated and 46 downregulated IRGs. Three IRGs (*S100A12*, *CCR7*, and *TNFSF4*) were identified as potential hub genes for prognosis. Those genes were subsequently subjected to the construction of the prognostic models. In the results, IPF patients categorized as high risk demonstrated a poor overall survival rate compared to patients categorized as low risk. Based on this prognostic model, the area under the curve (AUC) of the survival-dependent receiver operator characteristic (ROC) for 1-year, 2-year, and 3-year survival rates was 0.611, 0.695, and 0.681, respectively, in the GSE28042 cohort. These observations were validated in the GSE27957 cohort, confirming the good prognostic effect of this model. The expression of the three genes was validated in our cohort. We also conducted a nomogram based on the three IRGs’ mRNA for quantitative IPF prognosis.

**Conclusion:** Three IRGs (*S100A12*, *CCR7*, and *TNFSF4*) were identified as potential markers for the prognosis of IPF.

## Introduction

Idiopathic pulmonary fibrosis (IPF), caused by an unknown reason and characterized by poor clinical prognosis, is a progressive, chronic, and irreversible interstitial lung disease (ILD) (Raghu et al., 2011a; Raghu et al., 2015). IPF occurs in elderly and middle-aged adults, and the median survival time from diagnosis is 2–4 years (Ley et al., 2011). Due to the recent advantages in the development of new therapies, the prognosis of IPF has been improved. However, it remains incurable partly due to the complexity and incomprehension of the etiology (Nathan et al., 2016; Richeldi et al., 2016). Therefore, better understandings of the molecular mechanisms of IPF are vital for the improvement of the prognosis of patients with IPF, early screening, and diagnosis.

Historically, IPF is assumed to be caused by chronic inflammation. However, the significance of inflammation in the onset and development of IPF has been strongly challenged. Evidenced by poor response to anti-inflammatory therapy, inflammation has demonstrated to be a nonessential pathogenic event in IPF (Selman et al., 2001; Richeldi, 2013). However, inflammation presents at different stages of IPF as a secondary event due to the activation of the innate and adaptive immune system and participates in IPF development (Mura et al., 2005; Heukels et al., 2019). Gene variants in *TOLLIP* (Toll-interacting protein), directly involved in inflammatory processes, resulted in reduced protein expression, which possibly contributes to an increased pro-inflammatory response observed in IPF patients (Noth et al., 2013). So far, the pathogenic role of inflammation in IPF remains controversial.

Regarding the changes in the understanding of inflammation, some researchers suggest that glucocorticoid treatment, a classic anti-inflammation therapy, is potentially effective in selected IPF patients (Heukels et al., 2019; Khor et al., 2019). Recent advances in precision treatment and increasing awareness of patient subgroup classification indicate a fundamental role of inflammation in IPF (Zhang et al., 2015). Macrophage-targeted lung delivery of dexamethasone improves pulmonary fibrosis therapy *via* regulating the immune microenvironment (Sang et al., 2021). More understanding of inflammation in IPF provides more evidence for the anti-inflammation treatment of IPF patients. Therefore, a systematic functional study of inflammation-related genes (IRGs) in IPF is of critical importance in understanding the roles of inflammation in IPF.

The blood components, including peripheral blood mononuclear cells (PBMCs) and white blood cells, are indicative factors of disease onset, progress, and prognosis of IPF (Richards et al., 2012; Scott et al., 2019). Maya et al. identified a 52-gene signature in PBMCs to stratify IPF patients to different risk levels and validated this signature in a prospective cohort (Herazo-Maya et al., 2013; Herazo-Maya et al., 2017). Studies using large-scale IRG expression profiles of patients are critically important for stratifying IPF patients and predicting the prognosis of IPF with different inflammation degrees.

The subject of this study was to investigate the roles of inflammation played in IPF and explore the potential clinical utility of IRGs for prognostic stratifications. Therefore, we downloaded the mRNA expression data of PBMCs and the clinicopathological data from the Gene Expression Omnibus (GEO) repository. We identified abnormally expressed IRGs between IPF and control (CTRL) using bioinformatic analysis, and systematically investigated their potential molecular mechanisms and functions. Finally, we analyzed the potential impact of IRGs on prognosis by combining the IRG expression data with clinical information.

## Methods

### Data Acquisition and Processing

A total of 569 inflammation-related genes were extracted from the Gene Ontology and GO Annotations (https://www.ebi.ac.uk/QuickGO/), which provides detailed annotations of genes. We downloaded the microarray expression matrix dataset of 75 IPF and 19 CTRL PBMCs with corresponding clinical data from GSE28042 (https://www.ncbi.nlm.nih.gov/geo/query/acc.cgi?acc=GSE28042). To identify differently expressed inflammation-related genes between IPF and CTRL PBMCs, and the limma R package (http://www. bioconductor.org/packages) was used to perform the negative binomial distribution method according to the absolute value of fold change (FC) (>1.3) and false discovery rate (FDR) <0.05.

### Protein–Protein Interaction Network and Hub Inflammation-Related Gene Screening

The differently expressed IRGs were submitted to the Search Tool for the Retrieval of Interacting Genes (STRING) (http://string-db.org) (version 10.5) for the prediction of the PPI network. Meanwhile, Cytoscape (version 3.8.0) was applied to visualize the PPI networks. The hub genes were identified in the PPI network according to the prediction of cytoHubba plug-in with Nodes’ Scores. *p* values less than 0.05 were considered statistically significant.

### Inflammation-Related Prognostic Model Construction

Univariate Cox regression analysis and log-rank tests were applied to analyze the significant risk factors in all hub IRGs using the survival R package. Afterward, we set up a multivariate Cox regression model for the calculation of the risk scores to estimate the prognosis outcomes of IPF patients. Based on the above significant candidate hub genes, the risk scoring formula for each sample was as follows:

Risk Score = β1 * Exp1 + β2 * Exp2 + β3 * Exp3,

where *β* represented the coefficient value, while Exp represented the gene expression level.

Based on the median estimated risk score, IPF patients were categorized as high-risk and low-risk subgroups. The log-rank test was carried out for the comparison of the prognostic outcomes between the two subgroups. Further, the receiver operating characteristic (ROC) analysis was applied to assess the prognostic capabilities of the above model by the SurvivalROC R package.

### Validation of the Prognostic Model

45 samples of IPF patients with the corresponding prognosis from the GSE27957 dataset were used to validate the estimating results to evaluate the accuracy of the model prediction. We also performed expression validation of the three prognostic genes in our cohort by real-time PCR. We collected PBMC samples and clinical information of IPF patients in Tongji Hospital in Wuhan, China, from July 2020 to November 2020. Adult patients with clinical IPF diagnosis according to the American Thoracic Society/European Respiratory Society guideline (2011) were eligible for this study (Raghu et al., 2011b). This study was approved by the Ethics Committee of Tongji Hospital (IRB ID: 20150503). Results were presented as median with interquartile range (IQR), and nonparametric tests (Kruskal–Wallis or Mann–Whitney test) were used to compare across groups. Finally, a nomogram was conducted using the rms R package to predict the likelihood of a prognostic outcome in IPF patients. *p* values lower than 0.05 were set as statistical significance.

## Results

### Differentially Expressed Inflammation-Related Genes Identification

**FIGURE 1:**
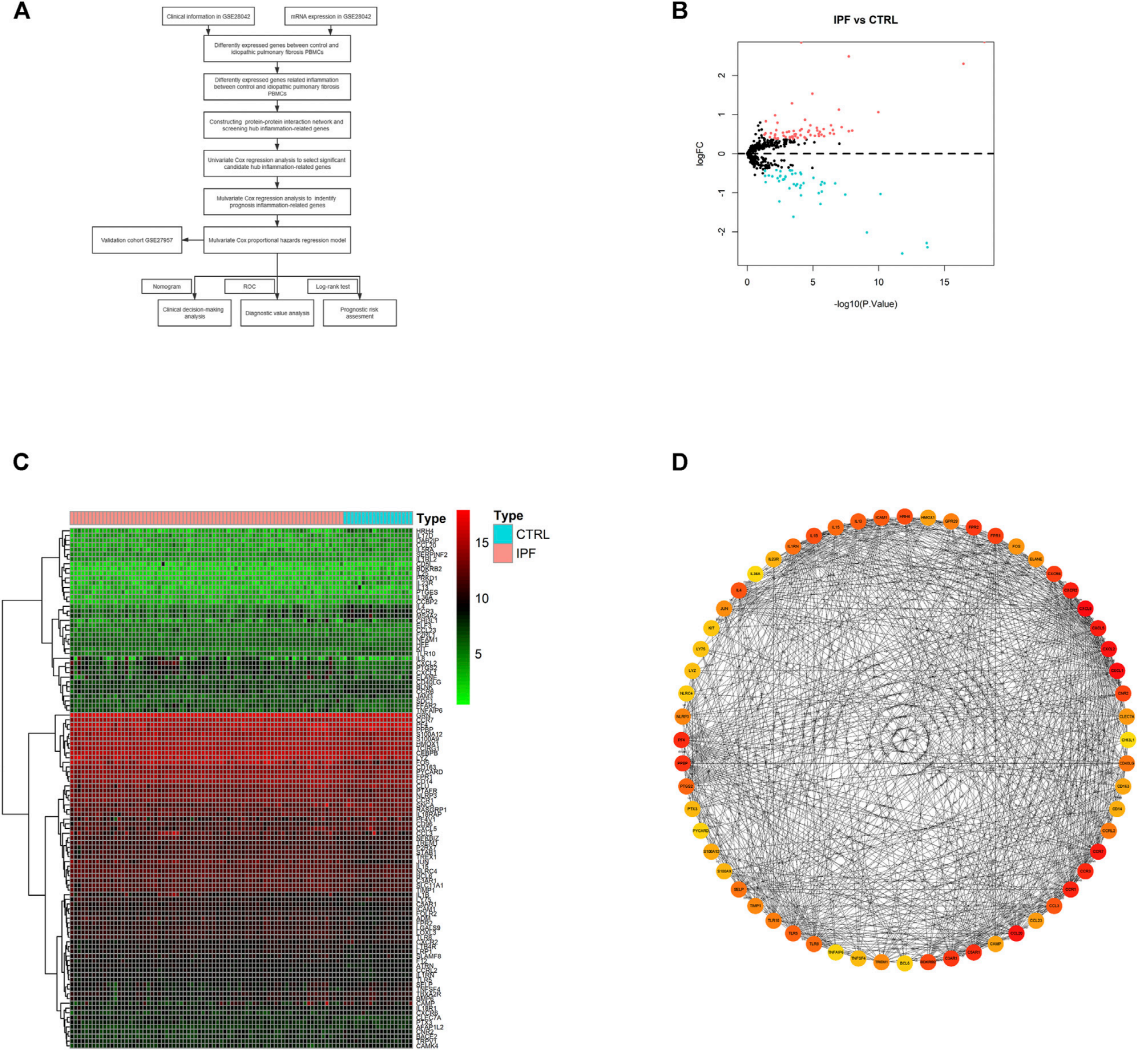
The differentially expressed inflammation-related genes (IRGs) in idiopathic pulmonary fibrosis. **(A)** Flowchart for analyzing inflammation signature associated with idiopathic pulmonary fibrosis. **(B)** Volcano plot and **(C)** heatmap of differentially expressed IRGs. **(D)** Top 60 hub genes from protein–protein interaction (PPI) network. Blue circles: downregulated genes with a fold change over 1.3, and red circles: upregulated genes with fold change over 1.3. Red circle: higher correlation, and orange circle: lower correlation.

A systematic analysis was carried out for the critical roles and the potential prognostic values of IRGs played in IPF. As shown in Figure 1A, the study was carried out according to the design. The databases of IPF patients were obtained from GEO-contained 75 IPF and 19 CTRL PBMCs samples. The R packages were used to process the data and discover differently expressed IRGs. According to the screening criteria, 110 IRGs were identified as differentially expressed between IPF and CTRL, containing 64 upregulated IRGs and 46 downregulated IRGs. The results were illustrated in the heatmap and volcano plot as in Figures 1B,C; details of the results were listed as in Supplementary Table S1.

### Protein–Protein Interaction Network Construction and Hub Gene Analysis

Gene network complexes were generated by STRING according to the results of differently expressed IRGs. The PPI network contained 110 nodes and 470 edges (Supplementary Figure S1). The interaction network was operated using cytoHubba tools in Cytoscape to identify top 60 hub IRGs (Figure 1D).

### Prognosis-Related Inflammation–Related Gene Selecting

**FIGURE 2:**
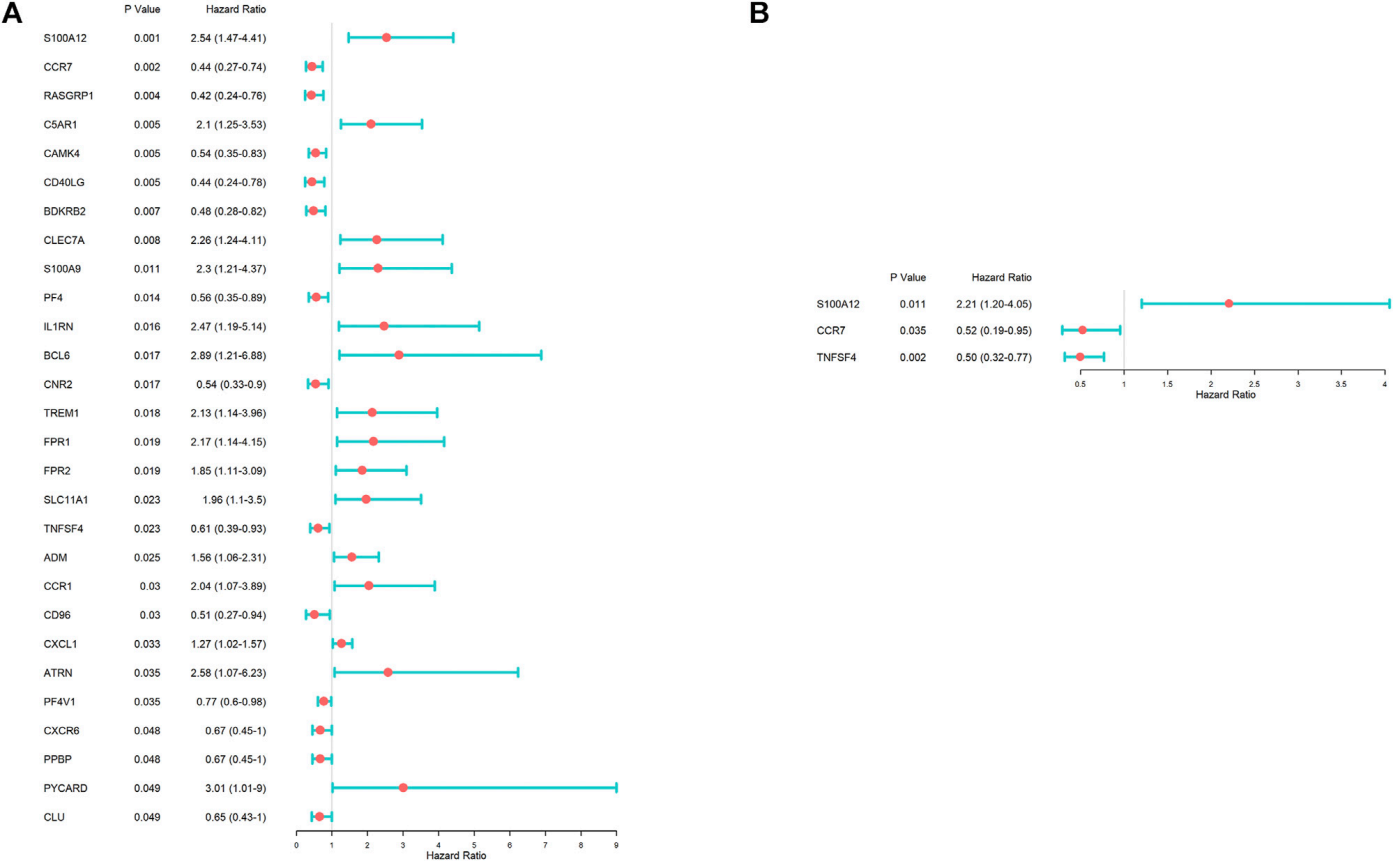
Identification of prognosis-associated inflammation-related genes according to Cox regression analysis. **(A)** Univariate Cox regression analysis. **(B)** Multivariate Cox regression analysis.

To identify IRGs of significant correlation with the overall survival, a univariate Cox regression analysis was established based on hub IRGs. As shown in the forest map of the hazard ratio (Figure 2A), 28 IRGs were significantly associated with the prognosis of IPF patients. To estimate the impact of prognostic-associated hub IRGs on the clinical outcomes and survival time, the 28 hub IRGs were further analyzed by multiple stepwise Cox regression. As shown in Figure 2B, S100 calcium binding protein A12 (*S100A12*), C-C motif chemokine receptor 7 (*CCR7*), and TNF superfamily member 4 (*TNFSF4*) were identified as independent predictors for IPF patients.

### Model Construction and Analysis for Prognosis-Related Genetic Risk Score

The three hub IRGs identified from the above analysis were used for the construction of the inflammation predictive model. The risk score of each IPF patient was calculated as

Risk Score = (0.792 * ExpS100A12) + (−0.645 * ExpCCR7) + (−0.704 * ExpTNFSF4).

**FIGURE 3:**
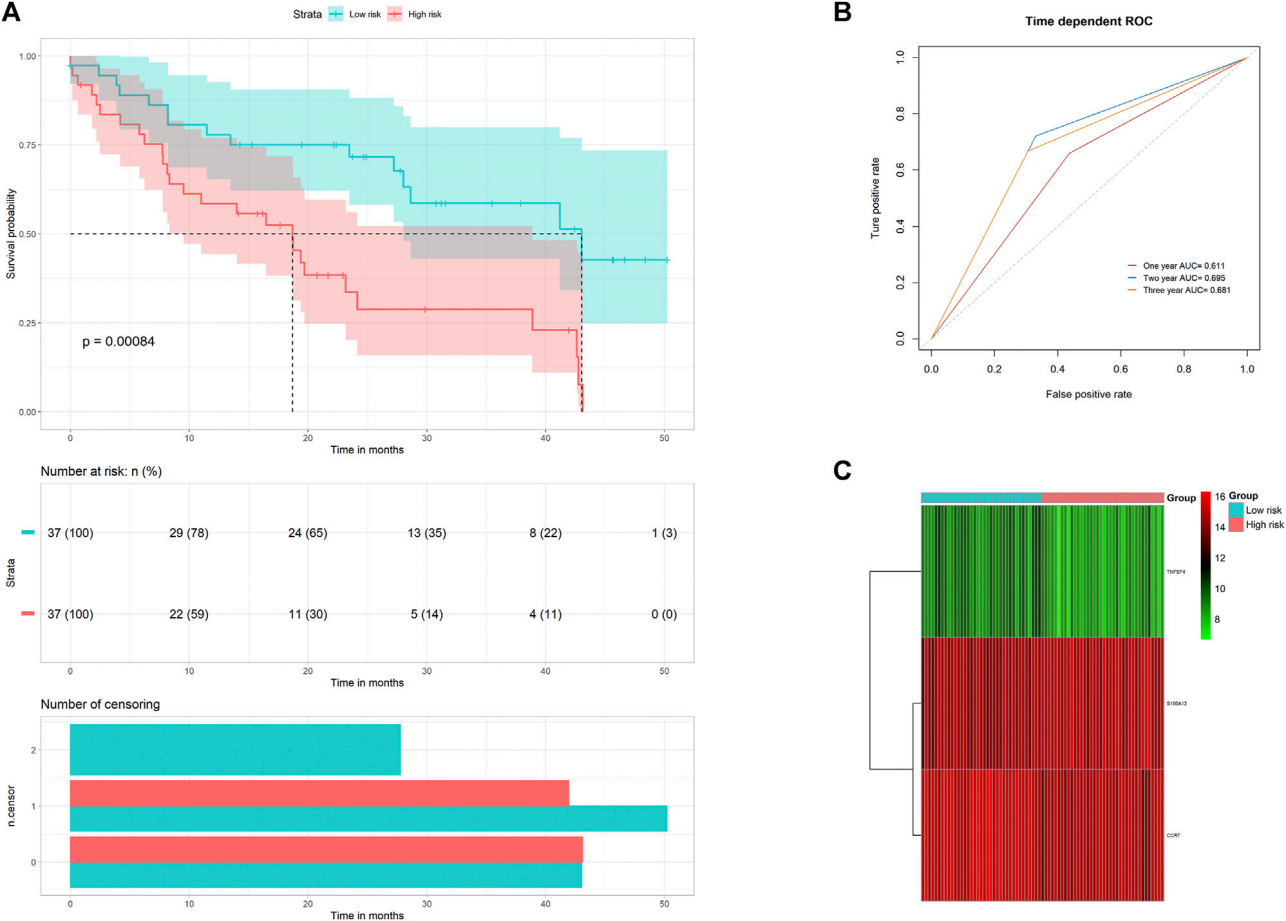
Risk score analysis of the three-gene prognostic model in the GSE28042 training cohort. **(A)** Survival curve for patients with low- or high-risk scores. **(B)** ROC curves for forecasting overall survival. **(C)** Heat map for gene expressions.

Then, we performed a survival analysis to evaluate the predictive effect of this model. As the result, 75 IPF patients were categorized as high-risk or low-risk according to the median of risk scores. As revealed by the result, IPF patients with high-risk scores were associated with poor prognosis compared with those with low-risk scores (Figure 3A). A survival-dependent ROC analysis was carried out for the assessment of the prognostic abilities (Figure 3B). The area under the ROC curve for 1 year, 2 years, and 3 years survival rates were 0.611, 0.695, and 0.681 respectively, indicating a moderate prognostic potential of IRGs in the survival prediction of the patients. The expressions heatmap of IRGs between subgroups are shown in Figure 3C.

### Validation of the Prognostic Model

**FIGURE 4:**
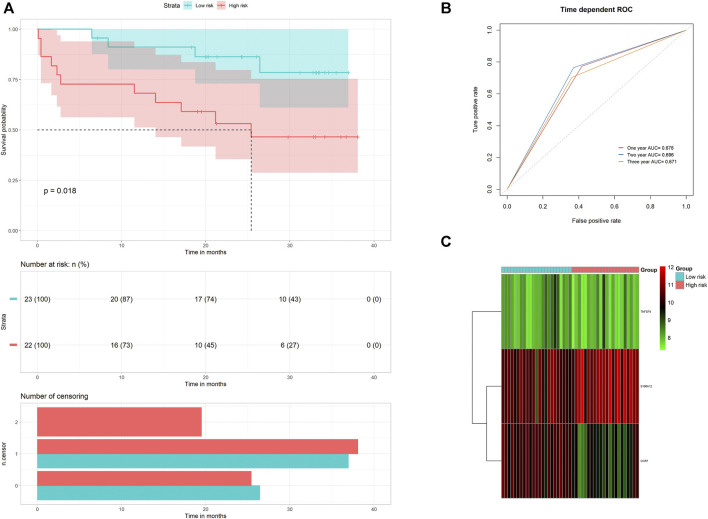
Risk score analysis of the three-gene prognostic model in the GSE27957 cohort. **(A)** Survival curve for patients with low- or high-risk scores **(B)** ROC curves for forecasting overall survival based on the risk score. **(C)** heat map for gene expressions.

**FIGURE 5:**
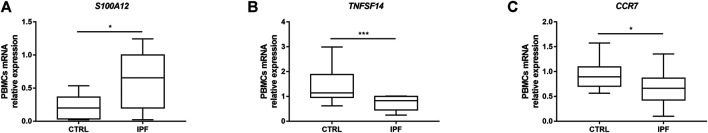
Changes in the expression of three prognostic genes in the peripheral blood mononuclear cells (PBMCs) of idiopathic pulmonary fibrosis (IPF) patients. Quantitative real-time polymerase chain reaction (qRT-PCR) was conducted to quantify the relative expression of the three prognostic genes in the PBMCs in healthy control (CTRL) (*n* = 16) and IPF (*n* = 14) patients. **(A)** SA100A12, **(B)** TNFSF4, and **(C)** CCR7. 18s RNA was used as an internal control gene. The data are presented as median with interquartile range (IQR) (Mann–Whitney test). ***p < 0.001, **p < 0.01, and *p < 0.05 were considered statistically significant.

On the one hand, to evaluate the prognostic accuracies of the three IRGs’ predictive model in IPF cohorts, this formula was applied to GSE27957 datasets. We discovered that high-risk patients had poorer overall survival than low-risk patients in GSE27957 cohorts (Figure 4). These results revealed that this prognostic model based on IRGs had better sensitivity and specificity. On the other hand, we performed verification of the three core genes expression in our cohort. As predicted, *S100A12* was significantly increased in IPF patients, while *CCR7* and *TNFSF4* were decreased (Figure 5).

### Construction of a Nomogram Based on Three Hub Inflammation-Related Genes

**FIGURE 6:**
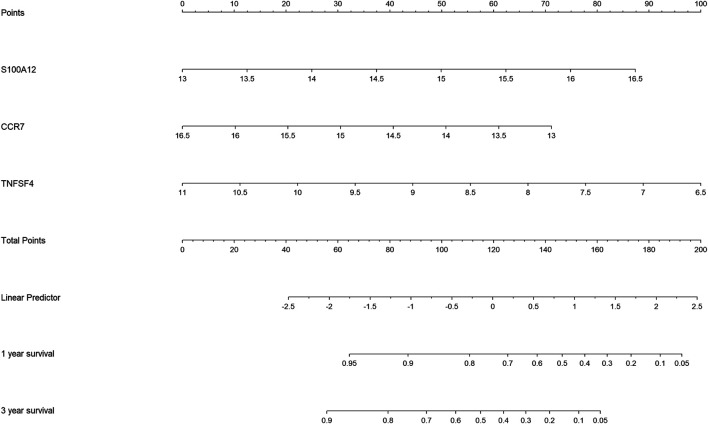
Nomogram for predicting 1- and 3-year overall survival of idiopathic pulmonary fibrosis (IPF) patients in the GSE28042 cohort.

For the establishment of quantitative methods for IPF prognosis, a nomogram was established according to the three hub IRGs (Figure 6). Points were distributed to each variable by the point scale in this nomogram based on the multivariable stepwise Cox regression analysis. We calculated the total points of each IPF patient by summing points of three variables. Next, we could infer the survival rate for IPF patients at 1 and 3 years by matching the total points with below survival rate in our nomogram.

## Discussion

As the contributions of inflammation in the development of IPF were complicated and controversial, a comprehensive analysis of IRGs has not been conducted to explore its clinical significance. From the perspective of inflammation, we identified and validated three prognostic IRGs. The results indicated that a prognostic model based on three IRGs can be applied for prognostic stratification in IPF patients, helping to provide individualized treatment based on patient risk.

In this study, we identified 110 differently expressed IRGs between IPF and CTRL PBMCs based on GSE28042 from GEO dataset, and constructed a PPI network and co-expression network of these IRGs. As a result, up to 60 hub IRGs were obtained. Many of the hub IRGs had been demonstrated to associate with the development and progression of IPF (Keane et al., 1997; Kowal-Bielecka et al., 2005; Okuda et al., 2015; Yang et al., 2018; Cui et al., 2020). Three IRGs were identified to associate with the prognosis of IPF, including *S100A12*, *CCR7*, and *TNFSF4*.

The increased expression level of *S100A12*, related to neutrophil recruitment and activation, was associated with significantly worse outcomes in IPF. A previous study has reported that *S100A12* levels in the lung, bronchoalveolar lavage fluid (BALF), and serum were increased in acute respiratory distress syndrome (ARDS) patients compared to healthy controls (Lorenz et al., 2008; Kikkawa et al., 2010). Thomas et al. have demonstrated that a high *S100A12* protein level in serum was associated with poor prognosis in the overall survival, transplant-free survival, and progression-free survival in IPF patients (Richards et al., 2012), which was consistent with our findings. *S100A12* could promote inflammation and cell apoptosis in sepsis-induced ARDS while inhibiting fibroblast migration *via* the receptor for advanced glycation end products (Tanaka et al., 2019; Zhang et al., 2020). The role of *S100A12* in pulmonary fibrosis is still worth investigating.

The decreased level of *CCR7* was related to IPF poor prognosis in this study. But Choi et al. (2006) reported that *CCR7* expression was significantly raised in usual interstitial pneumonia (UIP) relative to biopsies from patients diagnosed with nonspecific interstitial pneumonia (NSIP) or respiratory bronchiolitis/interstitial lung disease (RBILD). *CCR7* was expressed by IPF fibroblasts but not normal fibroblasts and could promote migration and proliferation of IPF fibroblasts (Pierce et al., 2007a; Habiel and Hogaboam, 2014). Therapeutic targeting *CCR7* abrogates pulmonary fibrosis induced by the adoptive transfer of human pulmonary fibroblasts to immunodeficient mice (Pierce et al., 2007b). The discrepancy between our result and the previous study might result from the important but opposing roles of *CCR7* in disease. The *CCR7* axis could combat the spread of cancer by trafficking of effecter cells involved in mounting an immune response to a growing tumor, while contribute to the expansion of cancer *via* controlling the migration of tumor cells towards the lymphatic system and metastasis (Salem et al., 2021).

The lower level of *TNFSF14* of PBMCs was associated with a worse IPF prognosis. The cytokine *TNFSF14* was demonstrated to be an important factor in the development of lung tissue remodeling in the murine model, which is associated with asthma, IPF, and systemic sclerosis (da Silva Antunes et al., 2018). Rana Herro et al. demonstrated that *TNFSF14* was upregulated in bleomycin-induced pulmonary fibrosis and is a profibrogenic cytokine (Herro et al., 2015). Considering that *TNFSF14* as an inflammatory indicator was increased in different diseases (Hsu et al., 2019; Fan et al., 2020), the serum protein level of *TNFSF14* should be validated in IPF patients.

Subsequently, a risk model was established for the prediction of IPF prognosis according to multiple stepwise Cox regression analysis of hub IRGs, validated using the GSE27957 cohort. In this study, the ROC curve analysis demonstrated that the IRG signature was better fitted for diagnosis rather than predicting poor prognosis in IPF patients. A nomogram was constructed for intuitively predicting the 1- and 3-year overall survival. This prognostic model based on these three IRGs is financially cost-less and efficient for clinical use.

Nonetheless, limitations were found in this prediction system. First, this prognostic model was constructed according to the GEO database; therefore, further validation in the clinical patient cohort was needed. Second, as a retrospective study, further prospective studies were needed for verification of the outcomes. Finally, functional experiments were needed to further reveal the potential mechanisms of IRGs in the future.

Conclusively, this study identified three IPF prognostic IRGs based on comprehensive bioinformatic analyses in GSE datasets. Our results contributed to the understanding of the roles inflammation played in IPF pathogenesis, and therefore potentially promoted the development of markers for prognosis assessment.

## Data Availability

The datasets presented in this study can be found in online repositories. The names of the repository/repositories and accession number(s) can be found in the article/Supplementary Material.

## Abbreviations

CCR7: C-C motif chemokine receptor 7, CTRL: control, FC: fold change, FDA: food and drug Administration, FDR: false discovery rate, GEO: gene expression omnibus, ILD: interstitial lung disease, IPF: idiopathic pulmonary fibrosis, IRGs: inflammation-related genes, PBMCs: peripheral blood mononuclear cells, PPT: protein–protein interaction, RA: rheumatoid arthritis, ROC: receiver operator characteristic, S100A12: S100 calcium binding protein A12, STRING: search tool for the retrieval of interacting genes, TOLLIP: toll-interacting protein, TNFSF4: TNF superfamily member 4.
