# Supplementary material for: Development and Validation of the Prognostic Index Based on Inflammation-Related Gene Analysis in Idiopathic Pulmonary Fibrosis
Source: Front Mol Biosci. 2021 Jul 22;8:667459. doi: 10.3389/fmolb.2021.667459 (PMC8339426; doi:10.3389/fmolb.2021.667459)
Supplement: Supplementary file 1 [file Table1.DOCX]

Supplemental Table 1: Differently expressed inflammation-related genes between idiopathic pulmonary fibrosis (IPF) and healthy control (CTRL).

| Gene Names | Log fold-change | P Value |
| --- | --- | --- |
| FOS | 2.856265 | 9.07E-19 |
| IL8 | 2.848641 | 7.89E-05 |
| CXCL2 | 2.488254 | 1.87E-08 |
| JUN | 2.299564 | 3.55E-17 |
| PTGS2 | 1.535018 | 1.10E-05 |
| CXCL1 | 1.288273 | 0.000395 |
| ADM | 1.128092 | 1.07E-07 |
| CLEC7A | 1.060239 | 1.06E-10 |
| CCL3 | 0.982729 | 0.007695 |
| FFAR2 | 0.866028 | 4.00E-05 |
| ELANE | 0.829562 | 0.038682 |
| IL1B | 0.790417 | 0.005037 |
| FPR1 | 0.725368 | 4.23E-07 |
| SLC11A1 | 0.724817 | 1.57E-05 |
| HMOX1 | 0.677137 | 6.52E-08 |
| NFKBIZ | 0.659738 | 5.03E-06 |
| ELF3 | 0.62908 | 7.24E-05 |
| F12 | 0.628324 | 1.53E-06 |
| LOXL3 | 0.606629 | 3.05E-07 |
| TNFAIP6 | 0.6018 | 0.006213 |
| S100A12 | 0.594658 | 9.50E-05 |
| LTB4R | 0.593773 | 4.69E-06 |
| CEBPB | 0.592464 | 1.04E-08 |
| CD14 | 0.590498 | 1.99E-05 |
| FPR2 | 0.579729 | 0.000292 |
| ICAM1 | 0.574625 | 1.89E-08 |
| STAB1 | 0.564678 | 0.000663 |
| NFAM1 | 0.558889 | 3.00E-06 |
| PTAFR | 0.557036 | 2.28E-05 |
| C5AR1 | 0.55258 | 0.000964 |
| SLAMF8 | 0.534633 | 0.001895 |
| LYZ | 0.524759 | 0.015769 |
| NLRC4 | 0.522853 | 5.04E-07 |
| CD5L | 0.521469 | 0.040468 |
| NLRP3 | 0.507983 | 2.58E-07 |
| IL15 | 0.507058 | 3.63E-06 |
| CCR1 | 0.502612 | 0.000206 |
| P2RX7 | 0.4941 | 0.00025 |
| LGALS9 | 0.491253 | 0.000218 |
| F2RL1 | 0.491019 | 0.007437 |
| TREM1 | 0.487052 | 0.000208 |
| ATRN | 0.486006 | 2.26E-06 |
| PTGES | 0.4859 | 0.049633 |
| TLR8 | 0.476771 | 1.52E-05 |
| IL1RN | 0.472788 | 8.74E-05 |
| SDC1 | 0.466784 | 0.007774 |
| C3AR1 | 0.458665 | 0.001707 |
| HFE | 0.455202 | 7.14E-06 |
| TCIRG1 | 0.449693 | 3.47E-06 |
| FOLR2 | 0.445597 | 0.000218 |
| PYCARD | 0.444638 | 1.41E-06 |
| S100A9 | 0.440172 | 0.000348 |
| TIMP1 | 0.431276 | 0.000413 |
| SERPINF2 | 0.424239 | 0.001202 |
| CCL23 | 0.423098 | 0.018682 |
| LRP1 | 0.422337 | 0.000564 |
| CCRL2 | 0.420148 | 4.38E-05 |
| TREX1 | 0.405685 | 0.000144 |
| CD163 | 0.405546 | 0.008158 |
| TLR5 | 0.396267 | 0.000574 |
| GRN | 0.390508 | 3.66E-05 |
| PTX3 | 0.389964 | 0.004632 |
| BCL6 | 0.385059 | 0.000323 |
| IL1RL2 | 0.384344 | 0.0064 |
| AFAP1L2 | -0.41836 | 0.000578 |
| IL36A | -0.419 | 0.006088 |
| RASGRP1 | -0.4306 | 0.000393 |
| TRPV1 | -0.43174 | 0.000535 |
| IL17D | -0.43933 | 0.04447 |
| CD40LG | -0.44607 | 0.002986 |
| LY75 | -0.48551 | 8.92E-05 |
| IL18R1 | -0.50335 | 0.000214 |
| CAMK4 | -0.52112 | 0.000377 |
| CD96 | -0.52155 | 8.08E-05 |
| JAM3 | -0.55907 | 0.022982 |
| BDKRB2 | -0.56789 | 0.003034 |
| IL13 | -0.56794 | 0.044285 |
| PRKD1 | -0.5752 | 0.003221 |
| SELP | -0.58014 | 0.012403 |
| IL18RAP | -0.58177 | 0.001236 |
| IL25 | -0.59707 | 0.00123 |
| CXCR2 | -0.61854 | 9.14E-06 |
| CLU | -0.62723 | 0.004865 |
| TBXA2R | -0.65285 | 0.001509 |
| DAB2IP | -0.66256 | 0.001878 |
| IL5RA | -0.70362 | 0.003792 |
| BLNK | -0.72278 | 2.05E-06 |
| PF4V1 | -0.72873 | 0.039813 |
| PF4 | -0.75403 | 5.11E-05 |
| CNR2 | -0.75523 | 1.31E-06 |
| CCR7 | -0.75877 | 2.04E-07 |
| CCBP2 | -0.77992 | 0.000161 |
| PPBP | -0.78169 | 0.000283 |
| BACE2 | -0.79287 | 2.46E-06 |
| CXCR6 | -0.80955 | 8.40E-05 |
| CCL20 | -0.81854 | 0.000195 |
| BMP6 | -0.87235 | 9.29E-05 |
| CXCL5 | -0.88517 | 0.000612 |
| TNFSF4 | -0.96954 | 2.14E-06 |
| CCR6 | -1.00745 | 3.62E-06 |
| KIT | -1.0321 | 7.24E-11 |
| TLR10 | -1.04725 | 3.49E-08 |
| SNCA | -1.06569 | 8.21E-05 |
| CAMP | -1.22065 | 0.003577 |
| IL23R | -1.28486 | 2.68E-06 |
| CHI3L1 | -1.61346 | 0.000308 |
| HRH4 | -2.01593 | 7.89E-10 |
| CCR3 | -2.28584 | 2.24E-14 |
| IL4 | -2.39365 | 1.95E-14 |
| MS4A2 | -2.55283 | 1.62E-12 |
