# Supplementary figures and images for: Development and Validation of the Prognostic Index Based on Inflammation-Related Gene Analysis in Idiopathic Pulmonary Fibrosis
Source: Front Mol Biosci. 2021 Jul 22;8:667459. doi: 10.3389/fmolb.2021.667459 (PMC8339426; doi:10.3389/fmolb.2021.667459)

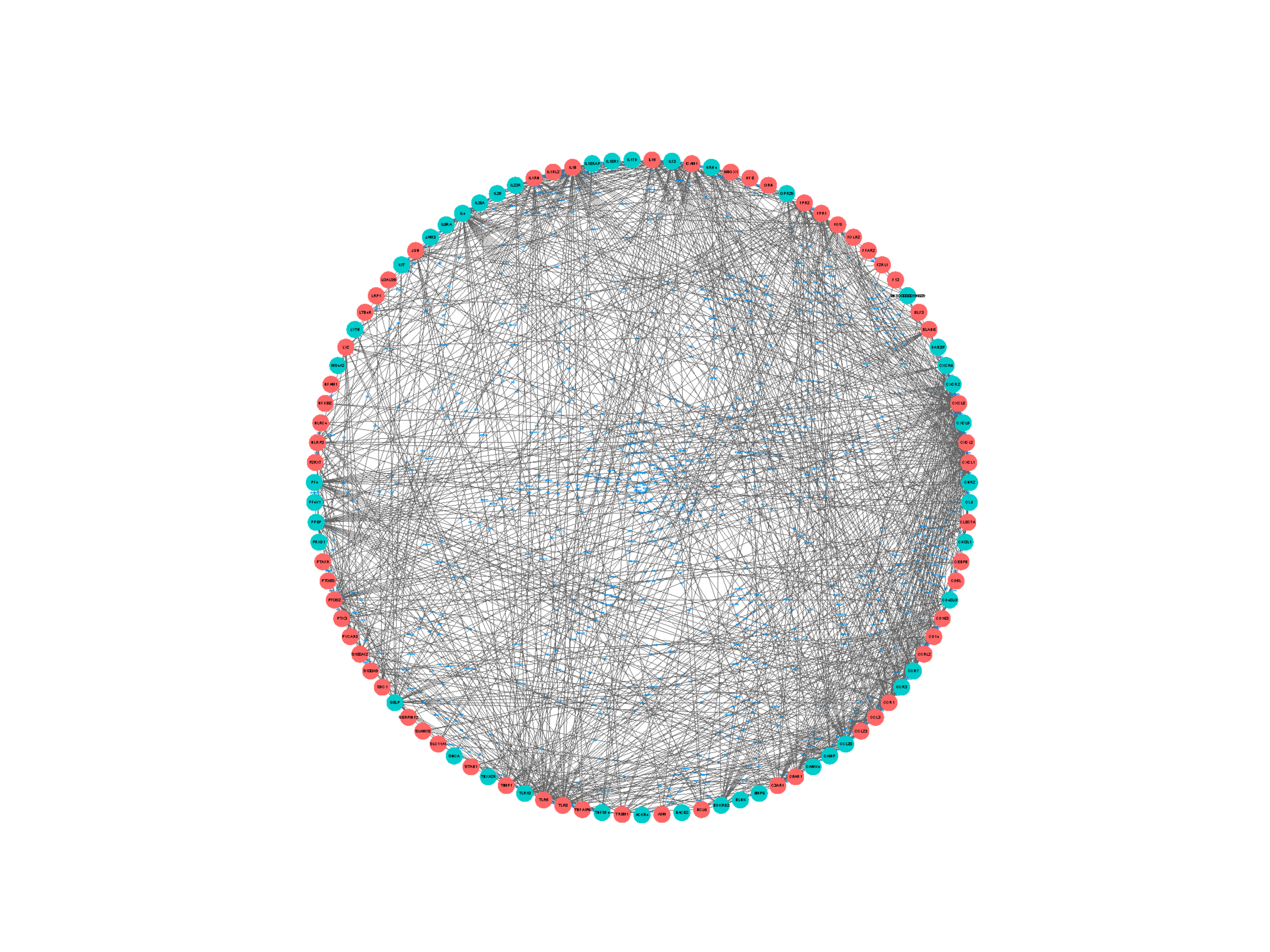

Supplement: Supplementary file 2 [file Image1.TIF]
