# Supplementary material for: Development and Validation of the Prognostic Index Based on Inflammation-Related Gene Analysis in Idiopathic Pulmonary Fibrosis
Source: Front Mol Biosci. 2021 Jul 22;8:667459. doi: 10.3389/fmolb.2021.667459 (PMC8339426; doi:10.3389/fmolb.2021.667459)
Supplement: Supplementary file 3 [file DataSheet1.docx]

**Supplemental figure 1.** Protein-protein interaction (PPI) network of differentially expressed inflammation-related genes.
